# Optimising Wheelchair Sprint Power Output Through Standardised and Individualised Force–Velocity Profiling

**DOI:** 10.1002/ejsc.70252

**Published:** 2026-09-15

**Authors:** Thomas Rietveld, Rowie J. F. Janssen, Thomas J. O'Brien, Pippa Bailey, Victoria L. Goosey‐Tolfrey

**Affiliations:** ^1^ Centre for Para and Disability Sport Innovation, School of Sport Exercise & Health Sciences Loughborough University Loughborough UK; ^2^ Department of Human Movement Sciences, Faculty of Behavioral and Movement Sciences Vrije Universiteit Amsterdam Amsterdam Movement Sciences Amsterdam the Netherlands; ^3^ University of Groningen University Medical Center Groningen Center for Human Movement Sciences Groningen the Netherlands

**Keywords:** disability sport, ergometry, exercise test, paralympics, propulsion technique, sports performance, strength, wheelchair sport

## Abstract

This study aimed to develop a standardised individualised wheelchair sprinting protocol with increasing resistances to generate linear force–velocity and parabolic power–velocity profiles, applicable across wheelchair sports with various classification levels. Twenty‐seven wheelchair athletes (16 wheelchair rugby [WR] and 11 wheelchair basketball [WB]) completed six 10 s sprints on a wheelchair ergometer with 2 min rest in between. Sprint 1 was completed at the same resistance coefficient for all athletes (μ = 0.012, court resistance). For the remaining five sprints, resistances were individually increased based on predicted peak power output values. Linear regression equations were determined between forces and velocities per push for both peak and mean values. Linear relationships were more stable using the mean values compared to peak values per push (*R* ^2^: 0.78 for mean power output and *R* ^2^: 0.72 for peak power output). Across the six sprints, forces increased (WR: +59%, and WB: +53%), whereas velocities decreased (WR: −40% and WB: −33%). The highest mean power output per push were achieved in sprint 3 for WR and sprint 4 for WB. On average, the peak of the power parabola occurred at a mean velocity of 2.85 ms^−1^ and mean power output of 414 W. This wheelchair sprint protocol with individualised increases in resistance provides an applicable force–velocity relationship across sports with various classification levels when modelled using the mean force and velocity per push. Together with the subsequent power–velocity relationship, this protocol can be used to optimise power production relative to individual baselines and help work towards the most optimal force–velocity profile.

## Highlights

- This study presents the first standardised individualised resistance‐based wheelchair sprinting protocol using an instrumented dual‐roller ergometer, applicable across wheelchair sports, sexes and classification levels.
- Linear force–velocity and parabolic power–velocity relationships were consistently obtained, with mean force–velocity measures providing greater robustness than peak values across increasing sprint resistances.
- Optimal power and velocity were highly individual and impairment‐specific, supporting the use of individualised profiling to guide force‐ or velocity‐specific training in wheelchair athletes.

## 1 Introduction

Wheelchair court sports (rugby, basketball and tennis) are characterised by repetitive short intermittent sprint bouts (van der Slikke et al. 2020; Rhodes et al. 2017). Sprint performance is affected by the athletes' impairment, therefore, understanding the determinants of successful wheelchair sprint performance is essential (Goosey‐Tolfrey et al. 2018). As outlined by the International Paralympic Committee (IPC), wheelchair sports employ a classification system to minimise the impact of impairment on the performance outcome in competition (Tweedy 2002; International Paralympic Committee (IPC) 2025). In wheelchair basketball (WB) and wheelchair rugby (WR), players are often divided into low‐point (LP) or high‐point (HP) players, based on severity of impairments, with HP players typically covering greater distances and achieving greater peak velocities on court (Rhodes et al. 2015). As these groups exhibit distinct wheelchair sprint performance profiles, it is essential to tailor training recommendations to the athletes' specific sport, physical capabilities and their role on court. This approach enhances individual performance, reduces injury risk and maximises the overall effectiveness of training interventions (Paulson and Goosey‐Tolfrey 2019).

To effectively tailor such training programmes, a deeper understanding of the mechanical and physiological factors underpinning sprint performance is essential. Wheelchair sprinting is a complex interaction between the athlete, wheelchair, configuration and the environment (Mason et al. 2013; Rietveld, Vegter, et al. 2021). Optimised (de)‐coupling and acceleration are key aspects across all wheelchair sports to increase power production and enhance overall performance (de Groot et al. 2017; Altmann et al. 2015). Sprint performance of wheelchair athletes is commonly assessed using over‐ground linear sprint tests over 5–20 m with timing gates and inertial measurement units (IMUs) (Gee et al. 2018; de Witte et al. 2018; Rietveld et al. 2019). Beyond sprint times, IMUs offer detailed push characteristics, such as frequency and cycle time, providing greater insights into wheelchair sprint kinematics (van der Slikke et al. 2016). However, despite efforts to predict power output on court, accurately measuring valid power output in wheelchair sports remains challenging (de Vette et al. 2023; van Dijk, Hoozemans, et al. 2024; van Dijk, Heringa, et al. 2024; Klimstra et al. 2023).

Lab‐based wheelchair ergometers remain the most reliable and controlled method to indirectly assess the power output in wheelchair athletes (de Klerk, Vegter, Goosey‐Tolfrey, et al. 2020). Standardised individualised protocols, such as the 10 s sprint and 30 s Wingate test, have been developed for wheelchair athletes (Janssen et al. 2025). Considering sprinting velocities and distances in wheelchair court sports, a 5–10 s sprint, on a realistic resistance, has the highest ecological validity (van der Slikke et al. 2020). The 30 s Wingate test measures maximal anaerobic power using a resistance that sufficiently limits velocities to produce higher forces and subsequently leads to maximal power output (Janssen et al. 2025; Bar‐Or 1987). Employing multiple sprints across resistance levels between sprint and Wingate resistances allows the investigation of propulsion technique over a range of forces and velocities (de Klerk, Vegter, Veeger, et al. 2020). This reveals an athletes' force–velocity generating capacity, guiding the athlete tailored strength or velocity–focused training. Although conventional methods exist in other sports, such as cycling, they differ in task mechanics and force application. Therefore, task‐specific multiload wheelchair sprint tests represent the established conventional method in this cohort.

During cyclic multijoint movements, force and velocity exhibit an approximately linear inverse relationship, reflecting biomechanical constraints between force generation and velocity (Jaric 2015; Hintzy et al. 2003). In WB, force and velocity showed strong correlations between on‐court sprints and laboratory‐based ballistic push‐off strength tests (Brassart et al. 2023). In WR, force–velocity relationships were explored using a 45m sprint on court using IMUs, with forces predicted from the IMU acceleration data (Klimstra et al. 2023). In the lab, a repetitive 5 × 15 s sprint test on a friction‐braked instrumented wheelchair ergometer provided the most ecologically valid analysis of the force–velocity relationship in WR players (Janssen et al. 2023). However, the study of (Janssen et al. 2023), used fixed resistances for all WR players resulting in underestimation of sprint power in several players, as shown by participants not achieving peak power, which limited validity (Janssen et al. 2023). Secondly, participants were largely restricted to male WR players (12 M/1F) leading to the exclusion of other wheelchair sports or female players which potentially exhibit distinct wheelchair sprinting profiles (van der Slikke et al. 2020; Rietveld et al. 2026). To address limitations of prior sprint protocols, resistances should be standardised and individualised to ensure each athlete performed sprints across their own power spectrum whilst keeping the total number of sprints constant.

Understanding force–velocity interactions across classification levels, sexes and sports offers coaches and practitioners insights into personalised training strategies, propulsion technique and wheelchair set‐up to maximise power output.^27^ A multisprint approach using individualised resistances would allow potential force–velocity profiling across an extensive group of wheelchair athletes. Therefore, this study aimed to develop a standardised and individualised wheelchair sprinting protocol using personalised resistance percentages. It focuses on generating linear force–velocity profiles and parabolic power–velocity profiles across wheelchair sports (WR and WB), sexes and various classification levels (LP and HP). It was hypothesised that each athlete could achieve their optimal power output regardless of sport or classification using this individualised approach.

## 2 Materials and Methods

### 2.1 Participants

Twenty‐seven wheelchair athletes participated in this study, 16 experienced WR players and 11 experienced WB players (Table 1). Players had a variety of impairment types, including spinal cord injury, cerebral palsy and amputations. Inclusion criteria were to play at a national level, with a minimum experience of 3 years. The study protocol was approved by the local ethical committee. All players provided written informed consent prior to participation in the study.

**TABLE 1:** Player characteristics.

	WR (*n* = 16)	WB (*n* = 11)
Male/Female	14/2	2/9
Age (years)	34 ± 7	26 ± 7
Body mass (kg)	69.8 ± 10.1	71.0 ± 17.5
Wheelchair mass (kg)	18.1 ± 1.6	12.1 ± 1.0
Total experience (years)	10 ± 5	10 ± 3
Weekly training (hours)	15 ± 5	13 ± 5
Classification		
0.5	2	NA
1.0	2	1
1.5	2	—
2.0	3	1
2.5	2	1
3.0	1	3
3.5	3	2
4.0	NA	1
4.5	NA	2

Abbreviations: WB, Wheelchair basketball; WR, Wheelchair rugby.

### 2.2 Equipment

All tests were performed in each player's own sports wheelchair on a Lode Esseda wheelchair ergometer (Lode, Groningen, the Netherlands). Tyre pressure was self‐selected and controlled (6–8 bars), with wheel sizes ranging from 24 to 27 inches, with camber angle ranging from 18° to 20°. The wheelchair ergometer, comprising two independent rollers, measured effective force and linear velocity at 100 Hz. Wheelchairs were strapped to the centre of the rollers, and an individualised calibration was completed to adjust motor resistance based on each wheelchair‐user combination. This procedure accounts for mass, distribution, alignment and tension on the fastening straps (de Klerk, Vegter, Veeger, et al. 2020).

### 2.3 Test Protocol

All players had previous experience pushing on a dual roller wheelchair ergometer. After completing the calibration procedure, players propelled their wheelchair for 2 min at a self‐selected pace as a warm‐up. Following a 2 min rest period, the main test protocol started.

Six 10 s sprints from a stationary start were completed with 2 min rest in between. The first sprint was completed at the same resistance coefficient for all players (μ = 0.012), replicating court resistance (van der Woude et al. 2003). For the remaining five sprints, resistances were individualised based on the predicted maximal power output for a 30s Wingate test (Equation 1). (Janssen et al. 2025) The mean velocity of sprint 1, mass of wheelchair‐athlete combination and wheelchair sport (WR or WB) were entered into the equation.

(Eq 1) $$P_{30}\;\left[W\right]=\left(-1.19+\left(1.02\;*\;V_{mean}\right)-\left(0.15\;*\;WR\right)-\left(0.26\;*\;WB\right)\right)\;*\;M_{total}$$

(Eq 2) $$\mu_{100\%}=\frac{P_{30}}{\frac{2\;*\;r_{w}}{r_{r}}\;*\;M_{total}\;*\;9.81}$$

(Eq 3) $$\mu_{25\%}=0.012+0.25\;*\;\left(\mu_{100\%}-0.012\right)$$

Sprint 5 was completed at the resistance with theoretically the highest chance of reaching maximal power output (Equation 2). Sprint 2 was at 25% of this resistance (Equation 3), sprint 3 at 50%, sprint 4 at 75% and sprint 6 at 125%. This range of resistances allows for an offset in case of under or overestimation. Fatigue had no effect on delivered power outputs across multiple short sprints in previous research (Janssen et al. 2023). As an example, if the predicted resistance coefficient of sprint 5 was 0.070, this would result in these six resistance coefficients (0.012, 0.026, 0.041, 0.055, 0.070 and 0.084).

### 2.4 Data Analysis

**FIGURE 1:**
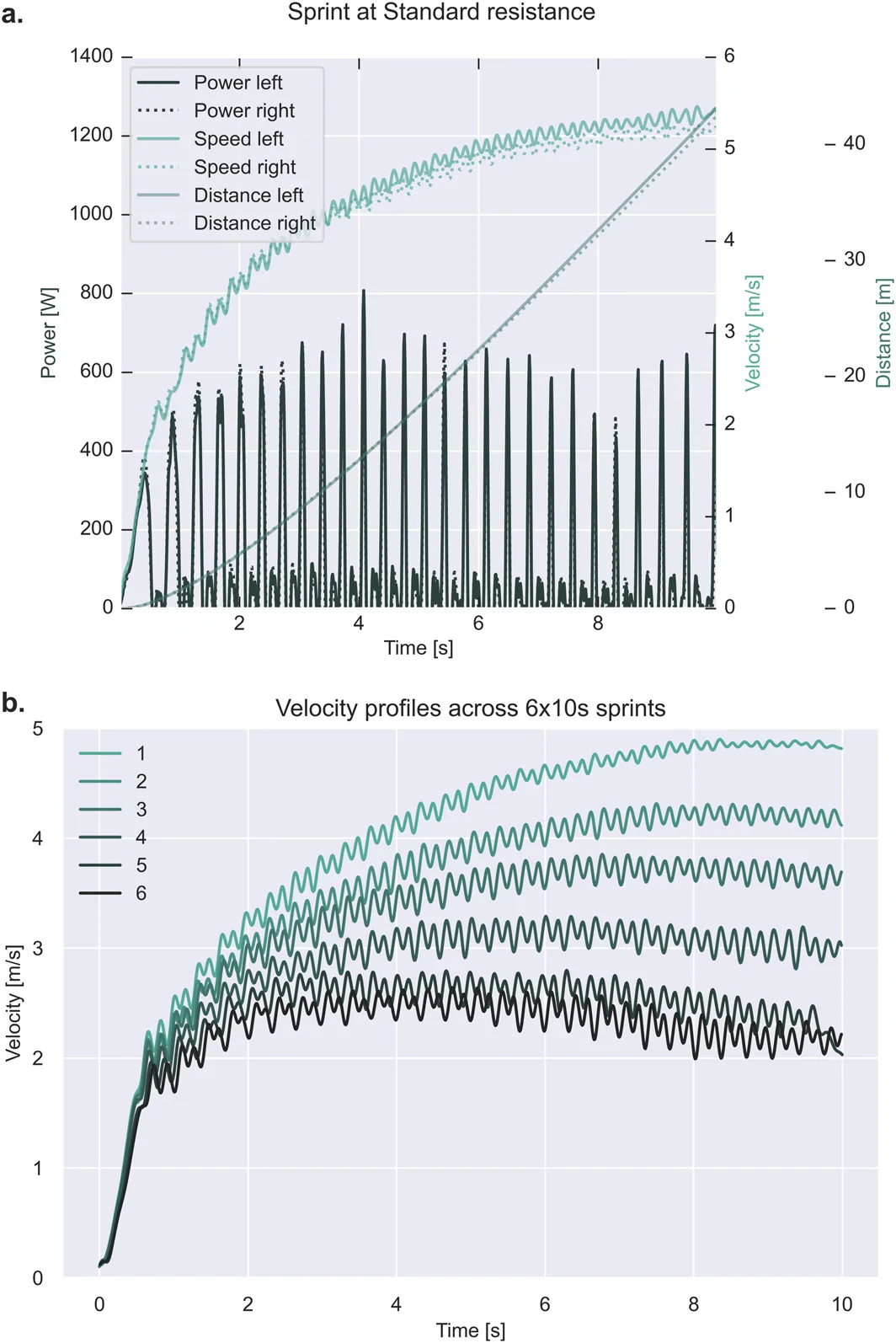
Visualisation of a 10s sprint at a standardised resistance (a) and velocity profiles across 6 × 10s sprints using individualised resistances (b).

All analyses were performed using custom‐written Python scripts (de Klerk et al. 2023). Force and velocity were directly measured via the two independent rollers of the ergometer, a reliable method validated against instrumented push rims (de Klerk, Vegter, Veeger, et al. 2020). Data were filtered using a second‐order Butterworth filter with a cut‐off frequency of 10 Hz for force data and 6 Hz for velocity data (de Klerk, Vegter, Veeger, et al. 2020). Power was determined through the multiplication of force and velocity. An example of the processed ergometer data can be seen in Figure 1. The figure visualises a sprint at standardised resistance (a) and a velocity profile across all sprints (b).

Sprint performance was calculated as distance covered and total work delivered (sum of left and right) over the completed 10s sprints. Push‐related outcomes were defined per push (Torque > 1Nm till torque < 1Nm) and averaged over all the pushes in a sprint (Vegter et al. 2014). For the peak power output (PO_peak_), peak force (F_peak_), mean power output (PO_mean_) and mean force (F_mean_), the sum of the left and right side was calculated. For the peak velocity (V_peak_), mean velocity (V_mean_)_,_ (neg) work, slope, push time and push frequency average of both sides were calculated.

### 2.5 Statistical Analysis

Normality of the data were checked using Shapiro–Wilk tests and visual inspections of the histograms and Q–Q plots. A mixed measures ANOVA was conducted to examine differences within sprints (1–6) and between sports (WR or WB). A Greenhouse Geisser correction was used to correct for sphericity. Partial eta squared effect sizes were reported and defined as small (≥ 0.01), medium (≥ 0.06) or large (≥ 0.14). (Cohen 1977).

Linear regression equations were determined between the F_peak_ and V_peak_ per push and F_mean_ and V_mean_ per push (excluding the first push, overcoming inertia). Velocity was used as the predictor and force as the dependent variable. Regression lines were extrapolated to get the full force–velocity spectrum and used to derive the theoretical peak force (F_0_; *y*‐intercept) and peak velocity (V_0_; *x*‐intercept). Force and velocity were multiplied to construct the power–velocity curve, from which the optimum power at optimum velocity was determined. Both peak and mean values were used to decide upon the best method to establish the force–velocity curves. The power–velocity curves were constructed from the linear regression lines using custom written Python scripts (de Klerk et al. 2023). The optimal velocity and subsequent power at this velocity were determined on this parabola. The force–velocity traces were individually investigated across sports (WR and WB) and classification levels (LP and HP).

## 3 Results

All WR and WB players successfully completed the individualised testing protocol to assess force–velocity relationships. Outcomes of all six sprints, including the multivariate ANOVA (Sprints: 1–6 and Sports: WB‐WR), are shown in Table 2.

**TABLE 2:** Sprint outcomes (mean ± SD) for the six 10s sprints, split per sport (WR and WB) including *p*‐values and effect sizes.

Sprint	Baseline		25%		50%		75%		100%		125%		p‐group	p‐sprint	Es sprint
Sport	WB	WR	WB	WR	WB	WR	WB	WR	WB	WR	WB	WR			
D [m]	29.70 (5.56)	31.90 (5.28)	28.03 (4.49)	29.19 (4.34)	25.83 (4.11)	26.60 (3.75)	23.86 (3.31)	24.05 (3.25)	21.56 (2.58)	21.69 (2.63)	19.93 (2.13)	19.49 (2.48)	0.70	< 0.001	0.89
W_total_ [J]	923 (259)	1072 (356)	1058 (286)	1255 (436)	1154 (325)	1391 (509)	1245 (381)	1515 (578)	1272 (399)	1559 (567)	1323 (424)	1604 (597)	0.17	< 0.001	0.72
PO_peak_ [W]	486 (133)	612 (283)	537 (156)	660 (323)	533 (159)	665 (332)	548 (173)	631 (315)	532 (172)	598 (303)	520 (159)	572 (299)	0.30	0.01	0.18
F_peak_ [N]	176 (31)	201 (67)	202 (38)	232 (86)	214 (39)	254 (97)	235 (50)	267 (104)	251 (58)	282 (114)	266 (59)	296 (120)	0.28	< 0.001	0.70
V_peak_ [m/s]	3.14 (0.59)	3.43 (0.54)	2.97 (0.49)	3.15 (0.45)	2.76 (0.45)	2.88 (0.40)	2.54 (0.35)	2.60 (0.34)	2.30 (0.28)	2.35 (0.28)	2.13 (0.23)	2.13 (0.27)	0.47	< 0.001	0.89
PO_mean_ [W]	286 (80)	350 (165)	311 (82)	373 (183)	320 (91)	384 (198)	331 (97)	371 (185)	321 (98)	360 (171)	312 (90)	353 (179)	0.34	0.03	0.14
F_mean_ [N]	107 (18)	117 (40)	121 (20)	133 (50)	132 (24)	149 (59)	146 (29)	160 (61)	156 (33)	172 (65)	164 (37)	186 (74)	0.37	< 0.001	0.76
V_mean_ [m/s]	3.10 (0.59)	3.39 (0.54)	2.92 (0.49)	3.10 (0.45)	2.71 (0.44)	2.82 (0.40)	2.49 (0.35)	2.53 (0.34)	2.24 (0.27)	2.28 (0.28)	2.07 (0.23)	2.05 (0.28)	0.52	< 0.001	0.90
W_push_ [J]	48 (15)	51 (14)	55 (16)	61 (15)	61 (17)	67 (18)	66 (20)	74 (18)	69 (21)	79 (19)	72 (24)	84 (22)	0.26	< 0.001	0.81
pt [s]	0.16 (0.03)	0.16 (0.07)	0.17 (0.03)	0.18 (0.07)	0.18 (0.02)	0.20 (0.09)	0.19 (0.03)	0.23 (0.10)	0.21 (0.03)	0.25 (0.10)	0.22 (0.03)	0.27 (0.11)	0.38	< 0.001	0.78
W_neg_ [J]	−10 (4)	−12 (6)	−10 (4)	−12 (7)	−9 (4)	−11 (7)	−9 (4)	−9 (6)	−8 (3)	−8 (5)	−7 (3)	−7 (5)	0.34	< 0.001	0.54
Slope [Nm/s]	674 (199)	867 (504)	742 (246)	949 (599)	764 (281)	1009 (653)	792 (355)	974 (694)	818 (376)	993 (749)	815 (349)	1017 (739)	0.48	0.04	0.11
Pf [#]	2.0 (0.3)	2.1 (0.5)	2.0 (0.3)	2.1 (0.5)	2.0 (0.3)	2.0 (0.5)	1.9 (0.3)	1.9 (0.5)	1.9 (0.3)	1.8 (0.5)	1.8 (0.3)	1.8 (0.5)	0.80	< 0.001	0.59
Mu [‐]	0.012 (−)	0.012 (−)	0.023 (0.005)	0.026 (0.005)	0.033 (0.011)	0.040 (0.009)	0.045 (0.016)	0.054 (0.013)	0.056 (0.021)	0.068 (0.018)	0.067 (0.026)	0.082 (0.022)	0.11	< 0.001	0.89

Abbreviations: D, distance; F, force; Mu, resistance coefficient; P, power output; Pf, push frequency; pt, push time; V, velocity; W, Work.

### 3.1 Linear Regression

**FIGURE 2:**
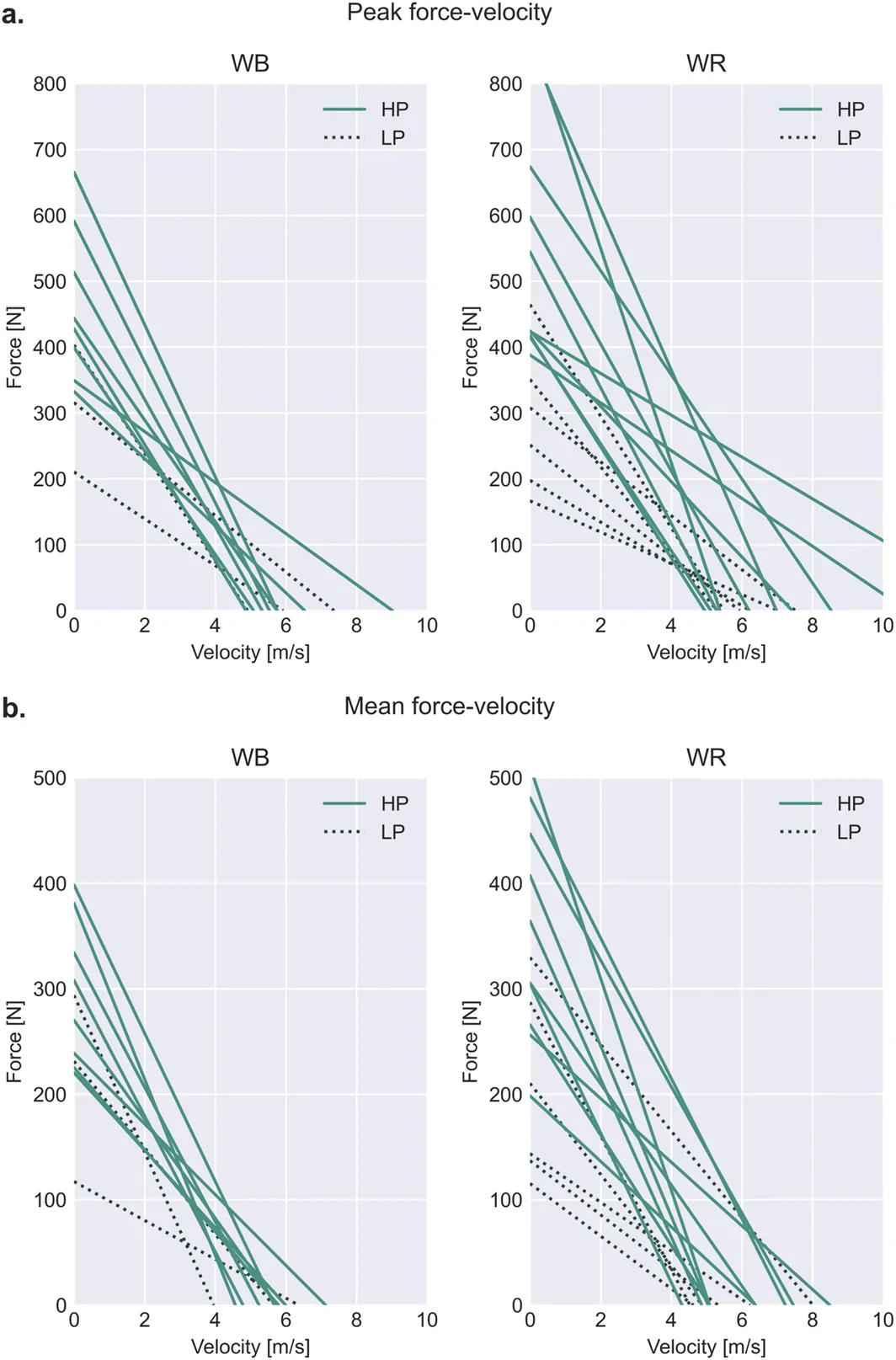
Linear regression force–velocity relationships for (a) peak (top) and (b) mean (bottom) values split for HP (high‐point) and LP (low‐point) for each sport WB (left) and WR (right).

Individual regression outcomes for peak (F_peak_ *x* V_peak_) and mean (F_mean_ *x* V_mean_) power are presented in Table 3 and Figure 2. Explained variances (*R* ^2^) were on average higher for mean values (*R* ^2^ = 0.78) than peak values (*R* ^2^ = 0.72) across both sports and classification levels (Table 3).

**TABLE 3:** Linear regression outcomes for peak force–velocity (left) and mean force–velocity (right) with mean per group and mean overall shown at the bottom of each section.

	Sport	*R* ^2^	Peak						Mean				
Class			V_opt_	PO_opt_	Y	X	#	*R* ^2^	V_opt_	PO_opt_	Y	X	#
LP	WB	0.63	2.93	311	210	5.92	4	0.53	3.15	186	117	6.36	—
LP	WB	0.65	2.43	495	403	4.91	5	0.78	1.96	290	294	3.95	6
LP	WB	0.79	3.72	581	316	7.36	2	0.88	2.85	326	231	5.64	4
LP‐mean	WB	0.69	3.03	462	309	6.06	4	0.73	2.65	267	214	5.32	3
HP	WB	0.84	2.64	683	513	5.33	4	0.91	2.26	435	381	4.57	6
HP	WB	0.67	3.23	543	333	6.53	3	0.82	2.87	326	225	5.79	4
HP	WB	0.88	2.85	957	665	5.75	6	0.94	2.81	564	398	5.67	6
HP	WB	0.87	2.84	637	444	5.75	5	0.88	2.85	388	270	5.75	5
HP	WB	0.74	2.79	817	591	5.53	4	0.79	2.59	438	334	5.24	5
HP	WB	0.89	2.46	521	428	4.87	5	0.90	2.42	368	308	4.79	5
HP	WB	0.80	2.52	507	399	5.08	6	0.69	2.97	330	220	5.99	3
HP	WB	0.72	4.46	787	349	9.02	2	0.76	3.52	425	239	7.12	5
HP‐mean	WB	0.80	2.97	682	465	5.98	4	0.84	2.79	409	297	5.62	5
LP	WR	0.69	3.08	308	198	6.23	2	0.88	2.69	182	137	5.32	3
LP	WR	0.75	2.94	373	251	5.93	1	0.50	3.08	224	144	6.23	1
LP	WR	0.82	2.62	464	351	5.29	3	0.87	2.41	256	115	4.62	4
LP	WR	0.47	3.48	292	167	7.02	—	0.78	2.34	133	210	4.87	4
LP	WR	0.52	2.71	637	464	5.48	5	0.82	3.96	660	329	8.01	5
LP	WR	0.86	3.72	578	308	7.51	1	0.90	2.24	325	287	4.53	2
LP‐mean	WR	0.68	3.09	442	290	6.24	2	0.79	2.79	297	204	5.60	3
HP	WR	0.55	5.40	1036	388	10.69	—	0.56	3.69	833	447	7.46	1
HP	WR	0.31	6.60	1412	424	13.33	—	0.75	4.20	544	256	8.49	1
HP	WR	0.79	3.75	787	424	7.42	2	0.59	2.51	338	266	5.08	3
HP	WR	0.83	2.45	516	417	4.95	6	0.90	3.16	487	305	6.38	6
HP	WR	0.68	2.61	719	544	5.28	6	0.72	2.49	513	407	5.04	6
HP	WR	0.79	2.53	531	415	5.12	4	0.69	2.46	443	364	4.86	6
HP	WR	0.90	2.72	1176	875	5.38	6	0.91	2.13	328	305	4.30	6
HP	WR	0.56	3.07	925	597	6.20	3	0.85	3.58	869	481	7.23	6
HP	WR	0.73	3.45	1493	855	6.98	3	0.69	2.52	649	511	5.08	3
HP	WR	0.77	4.22	1436	674	8.53	3	0.81	3.16	317	198	6.39	4
HP‐mean	WR	0.69	3.68	1003	561	7.39	3	0.75	2.99	532	354	6.03	4
All‐mean	WB‐WR	0.72	3.27	723	445	6.57	3	0.78	2.85	414	288	5.73	4

Abbreviations: Opt, optimal; PO, power output; V, velocity; X, X‐intercept; Y, Y‐intercept; #, Last sprint at which the optimal velocity was reached.

**FIGURE 3:**
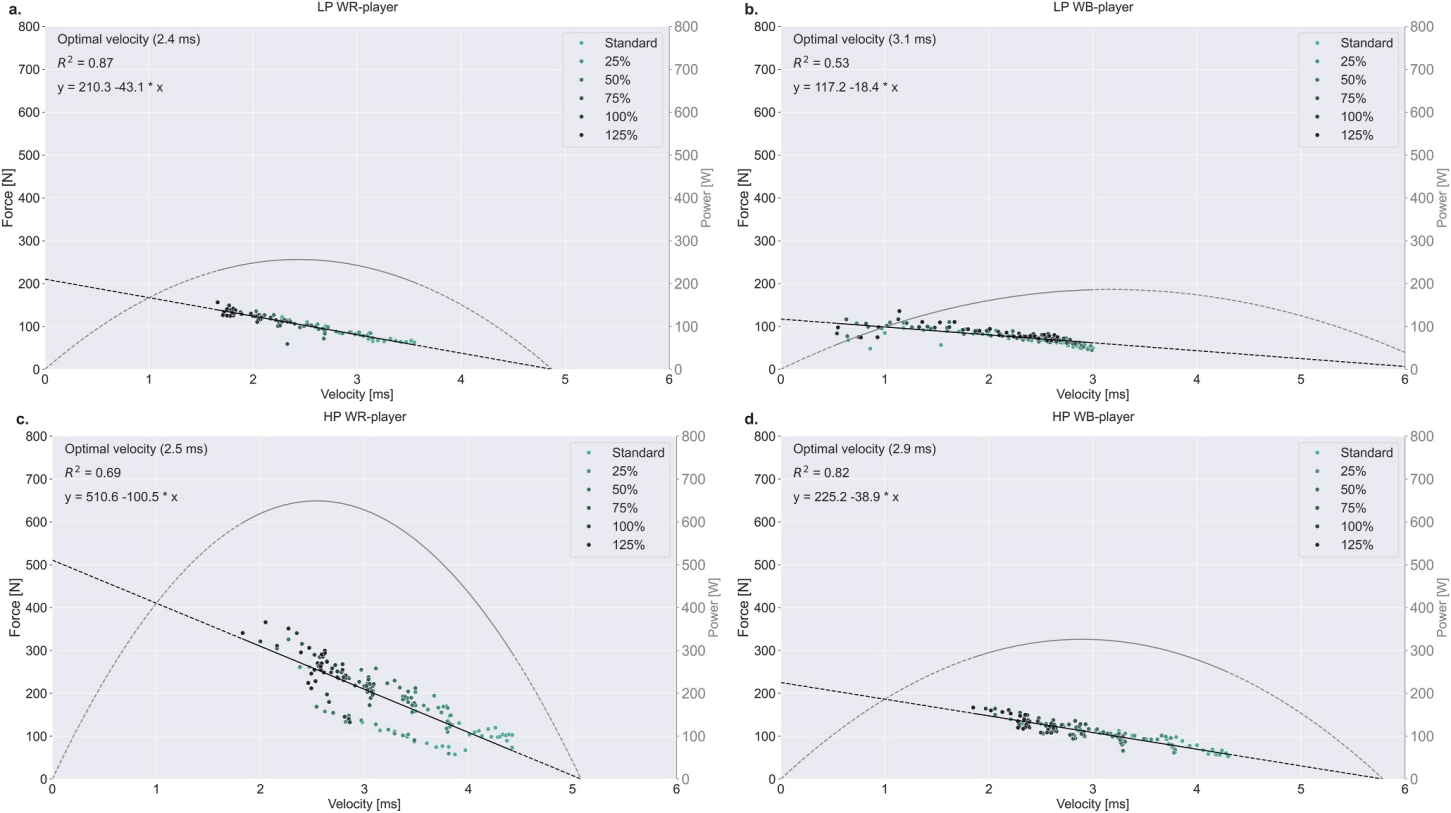
Four examples of individual force–velocity lines and power–velocity parabola. Forces and velocities are presented as mean values per push. The solid line represents the measured data, whereas the dotted lines are interpolated data. Data are presented of (a) LP‐WR player, (b) LP‐WB player, (c) HP‐WR player and (d) HP‐WB player. This shows the wide spectrum of individualised force–velocity profiles.

Four examples of power–velocity parabolas are illustrated in Figure 3. These highlight interindividual differences across sport and classification level, showing the wide range of force–velocity profiles. All athletes but one (26 out of 27) reached their optimal velocities and power outputs during the repetitive sprinting protocol as shown by the distinction between measured and interpolated data. All values for this LP‐WB player occurred before the top of the power parabola (Figure 3). For WB, optimal velocity was typically reached in sprint 4 (peak) and sprints 4–5 (mean). For WR, sprints 2–3 (peak) and sprints 3–4 (mean).

### 3.2 Sprint Performance

There was a significant main effect across all measured outcomes over the six sprints, with large effects sizes across all variables measured (Table 2). Distance, velocity (mean/max), negative work per push and push frequency decreased significantly from sprint 1 to sprint 6 (*p* < 0.001 and es > 0.54). In contrast, F_peak_, F_mean_, work and push time significantly increased (*p* < 0.001 and es > 0.70). Highest PO_mean_ and PO_peak_ per push were typically achieved in sprint 3 (50%) for WR and sprint 4 (75%) for WB (Table 2).

## 4 Discussion

The present study is the first to implement an individualised resistance‐based wheelchair sprinting protocol using an instrumented dual‐roller wheelchair ergometer, across different wheelchair sports (WR and WB), sexes, and various classification levels. The successful completion of the protocol by all athletes highlights its feasibility and practical relevance in applied sports settings. These findings build upon and extend the work of (Janssen et al. 2023), offering further support of the validity of force–velocity profiling in elite wheelchair athletes. In line with our hypothesis, using this individualised protocol, each athlete, except one, achieved the peak of the power output parabola. Decreases in V_mean_ and increases in F_mean_ were seen as sprint resistances increased across six sprints. This consistency across different sports (WR and WB) and various classification levels indicates that this standardised individualised protocol is effective for various sports and impairment types.

The approximate linear relationships between force and velocity per push were more stable using the mean values compared to peak values as outlined by greater *R* ^2^ values in all groups presented in Table 3. Mean values make use of the full propulsion cycle, whereas peak values only give a snapshot of the data, which is more prone to error. An average explained variance of 0.78 for the relation between F_mean_ and V_mean_ is suggestive of a strong linear relationship and in line with results from (Janssen et al. 2023) Previous research has identified a stronger linear relationship between force and velocity during wheelchair propulsion in nondisabled participants as outlined by the greater correlation coefficient (*r* = −0.94 ± 0.12) (Hintzy et al. 2003) However, nondisabled participants are typically more homogenous, which leads to less variability in the dataset. For example, at lower velocities, where mechanical demands and inertial forces are highest, athletes with reduced strength capacities or limited trunk function (e.g., spinal cord injury) may demonstrate increased push‐to‐push variability due to reliance on a smaller active muscle mass to generate the required high forces. Conversely, impairment types influencing coordination and timing (e.g., cerebral palsy) may influence variability at moderate and high velocities where coupling issues may occur (Ardekani et al. 2024; Veeger et al. 1991). These factors highlight the heterogeneity of movement patterns within this population and explain why natural deviations from the regression line may arise despite a fundamentally linear underlying force–velocity relationship.

The present study included all propulsion cycles in the analysis of each sprint which contrasts previous research and conventional F‐V methodologies where fewer data points likely decrease the coefficient of determination in the F‐V linear relationship (Jaric 2015; Hintzy et al. 2003). Inclusion of all propulsion cycles is inclusive of intersprint force and velocity changes whilst still aligning to correlation coefficients reported within other upper‐body force–velocity literature investigating multijoint noncyclic exercises (Jaric 2015). This does produce a substantially larger dataset that captures real push‐to‐push variability in propulsion biomechanics, bilateral asymmetries, hand rim contact mechanics, and upper = limb force application. As a result, our results produce more ecologically valid estimates of F_0_, V_0_, and optimal velocity which further improves the ecological validity of the protocol and allows more thorough training recommendations.

The current study found greater values for PO_peak_ per push, and F_peak_ per push compared to (Janssen et al. 2023), whilst including similar wheelchair athletes, suggesting a shorter sprint time of 10 s (instead of 15 s) did not compromise the player's ability to apply maximal force and power. Despite most players still accelerating within the 10s sprint duration, previous wheelchair‐specific research has highlighted PO_peak_ and V_peak_ is usually found within 10 s during a 30 s Wingate protocol performed at a relatively high resistance (O’Brien et al. 2025). Such findings justify the use of more ecologically valid 10 s force–velocity protocol at relative resistances (van der Slikke et al. 2020). In line with our hypothesis, PO_mean_ and PO_peak_ per push flattened off at higher resistances, indicating all players reached their true PO_mean_ and PO_peak_. This differed with earlier research that used fixed resistance increases (Hintzy et al. 2003; Janssen et al. 2023). Although decreases in velocity and increases in force were observed in previous studies with concomitant increases in PO, no flattening off in PO_peak_ was found suggesting optimal PO was not reached within the protocol. Adopting an individualised approach in the current study, PO_mean_ and PO_peak_ were observed in the third and fourth highest resistance sprint for WR and WB, respectively. Not being reliant on extrapolated values provides a more precise estimation of the players true force–velocity relationship.

From a sports perspective, a decline in PO was seen after sprint 3 in WR players and sprint 4 in WB players. The differences between sports are likely explained by wheelchair ergonomics, impairment types and sex. First, wheelchair sports often adopt different, individualised approaches to wheelchair setup to optimise the function of the player, alongside wheelchair mobility performance (Mason et al. 2013; Rietveld, Vegter, et al. 2021). Secondly, each sport has differing impairment criteria as outlined by the IPC (International Paralympic Committee (IPC) 2025). The nature of impairment influences the force and velocity generating capacities of players within similar classification groups as observed in previous studies (van der Slikke et al. 2020; Leicht et al. 2012). Thirdly, although our groups were divided by sport, they were dominated by the opposite sex, with recent research suggesting sex‐based differences having a significant influence on peak force production in wheelchair athletes (Rietveld et al. 2026; Mason et al. 2020). Despite the heterogeneity of our groups in sport, classification and sex, force–velocity curves could be constructed for every individual (*R* ^2^ = 0.50–0.91). Individualised force–velocity profiling enables the prescription of strength training interventions to enhance adaptations for force generation or enhanced velocity capabilities.

### 4.1 Future Recommendations and Limitations

The current research presented a standardised, individualised and easy to apply method to assess force–velocity profiling in wheelchair sports. Each athlete in our study displayed a unique force–velocity relationship, resulting in individualised regression equations (i.e., subject specific slopes and intercepts). Parameters are influenced by multiple factors (e.g., impairment type, propulsion technique, strength capacity and wheelchair configuration), a universal prediction model would not accurately represent the heterogeneous profiles observed (Janssen et al. 2022, 2025). As an optimum, players need to work towards the best possible model fit as well as improve optimal power production compared to their own baseline measure. Force–velocity profiling could be used as a monitoring tool for coaches within sessions or across a season. Using an individualised force–velocity protocol after a block of strength or velocity specific training, would give insights into progression of power production on an individual or group level.

The current study only included WB and WR athletes, but the presented protocol might be relevant for other wheelchair sports where sprinting is essential, such as wheelchair tennis or wheelchair racing. In wheelchair tennis, there is an additional constraint of a tennis racket influencing the sprint performances of a player (de Groot et al. 2017; Goosey‐Tolfrey and Moss 2005). With the rollers of the ergometer acting independently, bilateral symmetry can be further explored (Goosey‐Tolfrey et al. 2018; de Klerk, Vegter, Veeger, et al. 2020). For wheelchair racing, the use of a smaller hand rim design limits their acceleration potential, whilst being able to reach velocities up to 9 m/s suggestive of the need for longer sprint durations and less repetitions in this population.

The current protocol was individualised by a standardised resistance in the first sprint for every player, set at 0.012, compared to a deceleration test used in previous work (Janssen et al. 2023). A standardised value was chosen to create a similar baseline and accommodate various wheelchair sports, with a range of floor types encountered. Although 0.012 (μ) is used for a gym court in WB, wooden spring WR courts might be lower, at 0.008–0.009 (μ), based on anecdotal evidence. Wheelchair tennis is also played on various surfaces, with hardcourt (μ = 0.008), clay (μ = 0.012) and grass (μ = 0.040) showing clear differences in rolling resistance through a case study (Rietveld, Mason, et al. 2021). As a result, future research should investigate an additional sprint at a lower resistance to increase ecological validity. This would also help uncover the full potential of players that are more velocity driven such as those with limited trunk function, such as the LP‐WB player in this study not reaching peak power values. Previous research suggests trunk function impacts wheelchair sprint performance (Altmann et al. 2015), in particular accelerating the wheelchair which subsequently influences the individualised resistances for the remaining sprints within the proposed protocol.

## 5 Perspective

The current research presented a standardised and individualised resistance‐based wheelchair sprinting protocol on a wheelchair ergometer for wheelchair athletes. Linear force–velocity and parabolic power–velocity profiles could be constructed across wheelchair sports (WR and WB) and various classification groups. The linear relationships between force and velocity per push were more stable using the mean values per push compared to peak values. Optimal power and velocity values are specific for every individual with various impairment types. The presented individualised approach enables practitioner to use the equations presented in this study to establish individualised protocols to determine the force and velocity generating capacity of their athletes. This can guide training programmes to subsequently improve optimal power production and to assess training responses, focusing on specific training for force generation or enhanced velocity capabilities.

## Funding

This project was supported by the Peter Harrison Centre for Disability Sport, Loughborough University.

## Ethics Statement

Ethical approval was granted by the Loughborough University ethics committee (1271).

## Conflicts of Interest

The authors declare no conflicts of interest.

## Data Availability

Data are available on request from the corresponding author. Data are not publicly available due to privacy or ethical restrictions.
